# Seroepidemiological survey on pigs and cattle for novel K88 (F4)-like colonisation factor detected in human enterotoxigenic *Escherichia coli*

**DOI:** 10.1017/S0950268821002697

**Published:** 2021-12-13

**Authors:** Yoshihiko Tanimoto, Miyoko Inoue, Kana Komatsu, Atsuyuki Odani, Takayuki Wada, Eriko Kage-Nakadai, Yoshikazu Nishikawa

**Affiliations:** Department of Food and Human Health Sciences, Graduate School of Human Life Science, Osaka City University, Osaka, Japan

**Keywords:** Cattle, enterotoxigenic *Escherichia coli*, K88 antigen, pig, seroepidemiology, zoonosis

## Abstract

Enterotoxigenic *Escherichia coli* (ETEC) strains that express various fimbrial or nonfimbrial colonisation factors (CFs) and enterotoxins are critical causes of diarrhoeal diseases. Human ETEC serotype O169:H41 (O169) has been a representative of epidemic ETEC worldwide; the organism shows massive adherence to HEp-2 cells similar to enteroaggregative *E. coli*. Previously, we determined the complete sequence of the unstable virulence plasmid, pEntYN10. The plasmid included a unique set of genes encoding a novel CF resembling K88 (F4) of porcine ETEC, in addition to CS6, a well-known representative CF of human ETEC, and another novel CF similar to CS8 (CFA/III) of human ETEC. In the present study, we focused on K88-like CF (hereafter, K88_O169_) that may allow the organisms to infect domestic livestock like original K88-harbouring strains that can cause diarrhoea in piglets. Samples were tested for antibodies against recombinant proteins of possible paralogous adhesins, FaeG1 and FaeG2, from K88_O169_ and the FaeG of typical K88 (F4). The seroepidemiological study using recombinant antigens (two paralogs FaeG1 and FaeG2 from K88_O169_) showed reactivity of porcine (18.0%) and bovine (17.1%) sera to K88_O169_ FaeG1 and/or FaeG2 antigens on indirect ELISA tests. These results suggest that *E. coli* with K88_O169_ adhesin can infect various hosts, including pigs and cattle. This is the first report of domestic livestock having antibodies to K88_O169_ of human ETEC. Although human ETEC had been thought to be distinguished from those of domestic animals based on CFs, zoonotic strains may conceal themselves among human ETEC organisms. The concept of One Health should be adopted to intervene in ETEC infections among animals and humans.

## Introduction

Enterotoxigenic *Escherichia coli* (ETEC) is a diarrhoeagenic *E. coli* that causes diarrhoea not only in humans but also in various animals, such as pigs, cattle and sheep [1]. Regardless of host, colonisation of the intestinal epithelia is an essential first step in the pathogenesis of ETEC infection mediated by a variety of colonisation factors (CFs) [2]. Subsequently, ETEC secretes host-damaging toxins such as heat-labile (LT) and/or heat-stable (ST) enterotoxins, which give rise to intestinal symptoms such as diarrhoea [3, 4].

ST-producing ETEC O169:H41 (O169) was first identified as an aetiological serotype in human foodborne cases in Japan [5]. Previously, we reported that the O169 strain YN10 harbours the unstable large plasmid pEntYN10, which codes for genes of three CFs, CS6 and two novel CFs resembling CS8 (so-called CFA/III) and F4 (so-called K88). The operon coding the novel K88-like CF (hereinafter, K88_O169_) has two paralogous major adhesin-like subunits, *faeG1* and *faeG2*, which have 37–44% amino acid homology with *faeG* of the original K88 [6].

K88 and its related CFs have been found with bacteria isolated from a variety of hosts. K88 was initially identified as a CF of swine ETEC [7, 8], whereas CS31A was reported as a K88-related CF from bovine ETEC [9]. Thus, *E. coli* strains with analogous CFs were isolated from multiple hosts, suggesting that K88-related CFs can lead to colonisation in various host species. Furthermore, an ETEC/Shiga toxin-producing *E. coli* (STEC) hybrid strain was recently isolated from a patient with hemolytic-uremic syndrome (HUS); the organism possessed an F4-like adhesin of the protein sequence that was similar to CS31A of bovine ETEC [10]. The presence of multiple CFs in O169 may indicate the potential for multi-host transmission: a complex zoonotic network may be supported, and this contradicts the one-to-one host-pathogen relationship that has been commonly accepted for ETEC.

This study examined the seroepidemiological status of livestock against K88_O169_ as a preliminary investigation to test this hypothesis. Antibody titres against K88_O169_ of pigs and cattle reared in various areas in Japan were examined by an indirect ELISA method using recombinant proteins of the adhesins, FaeG.

## Materials and methods

### Sample collection

In this study, sera of 200 pigs and 105 cattle collected from June 2019 to March 2020 were kindly provided by the Osaka City Meat Hygiene Inspection Center for serological assays. This facility is partnered with the slaughterhouse and is responsible for hygiene inspections of meat supplied to the metropolitan area and also conducts research on livestock animals transported from a wide range of regions in Japan. Breeders and the prefectures where the animals were raised are listed in Supporting Tables S1 and S2.

### Recombinant proteins

Recombinant proteins were created using the pET expression system (Novagen). The codon-optimised typical *faeG* gene of the original K88 (K88ac variant, accession ID: AJ616239) [11] was synthesised by Eurofins Genomics KK (Tokyo, Japan). *faeG1* and *faeG2* genes of K88_O169_ were amplified by PCR with primers for the O169 plasmid pEntYN10 [6] as shown in Supporting Table S3. The primers were designed to remove signal peptides predicted using SignalP 5.0 web-based resource [12] from the recombinant proteins. These gene products and pET30a vector (Novagen) were digested with *Sal*I and *Not*I restriction enzymes and ligated. The resulting plasmids (pET30a-*faeG*, pET30a-*faeG1* and pET30a-*faeG2*) were recovered by transformation into *E. coli* BL21 (DE3) with selection for kanamycin resistance. After the bacteria were grown at 37 °C for 3 h, protein expression was induced with 1 mm of isopropyl-*β*-D-thiogalactopyranoside (IPTG; Takara Bio) at the final concentration at 25 °C. After overnight incubation, the cells were solubilised with 8 M urea. The recombinant protein was purified using the His60 Ni gravity column purification kit (Takara Bio) according to the manual instruction. Finally, the solution was exchanged with phosphate-buffered saline (PBS) using Amicon Ultra-15 10 K Centrifugal Filter Devices (Merck). Purity was confirmed by the absence of contamination bands on SDS-PAGE.

### Antiserum

Antiserum to the original K88 was purchased from Denka Seiken (*E. coli* K88 rabbit antiserum). Rabbit polyclonal antibodies against FaeG1 and FaeG2 developed by Eurofins Genomics Antibody Service were used as positive controls.

### Indirect ELISA

Sample sera were examined for the antibodies against FaeG of K88 and FaeG1 and FaeG2 of K88_O169_ with indirect ELISA methods. Purified protein was coated on 96-well half-area plates (Corning) at 0.1 μg/ml with coating buffer (13 mm Na_2_CO_3_, 35 mm NaHCO_3_) overnight at 4 °C. After washing three times with PBS-T (PBS containing 0.05% Tween-20), plates were blocked by adding blocking buffer (0.4% Block Ace, Dainippon Pharmaceutical) and incubated for 1–2 h. After three washes, sample sera (1:200 dilution in PBS-T) and antibodies (1:1000 dilution) as a positive control were added to the plate and incubated for 1 h. After three washes, a 1: 30 000 dilution of the secondary antibody solution was added to each well and incubated for 1 h. The secondary antibodies used for ELISA were as follows: HRP-linked anti-whole rabbit IgG donkey serum (GE Healthcare, #NA934) for detection of the positive control, HRP-linked anti-pig whole IgG goat antibodies (Abcam, #ab6915) for porcine samples, and HRP-linked anti-cow whole IgG goat antibodies (Abcam, #ab102154) for bovine samples. After three washes, TMB substrate (TMB Microwell Peroxidase Substrate System, KPL) was added to the plates, incubated for 5 min in the dark, stopped with 1 M H_2_SO_4_, and measured at wavelengths of 450 nm/550 nm with a microplate reader (Wallac 1420 ARVOsx, Perkin Elmer). Sample to positive (S/P) ratio (%) was calculated as follows: (Sample serum – blank)/(Positive control antibody – blank). S/P ratio >0.4 was considered as positive [13, 14].

### Statistical analysis

The results were statistically analysed using Prism software (GraphPad, ver. 8.4.3). Fisher's exact test was employed to assess the association between porcine and bovine seroprevalence.

## Results

**Fig. 1.**
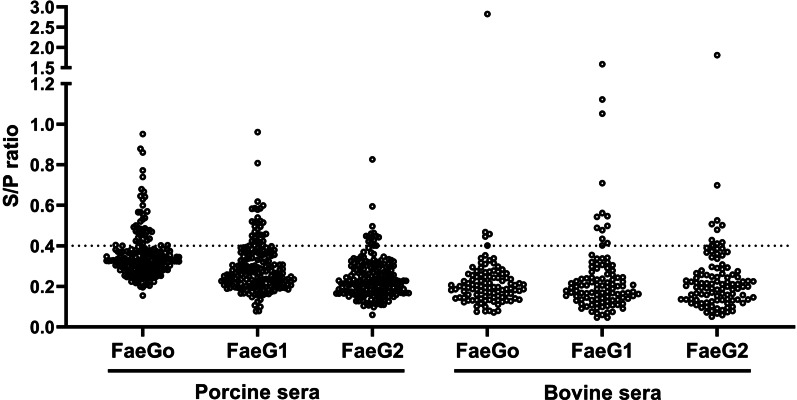
Indirect ELISA plot of porcine and bovine sera. S/P ratio for individual porcine and bovine sera against FaeG1, FaeG2 and FaeG_O_.

Anti-FaeG1 and -FaeG2 antibodies in porcine and bovine sera were measured to determine whether K88_O169_ may be another CF for infection of pigs or cattle. Among the samples tested, a portion of the porcine (18.0%, 36/200) and bovine (17.1%, 18/105) sera reacted to one or both of FaeG1 and FaeG2 antigens of K88_O169_ (Table 1, Fig. 1). The number of the 54 positive sera samples reacting to FaeG1, FaeG2, and both antigens was 29 (54%), 9 (17%) and 16 (29%), respectively. As 45 and 25 sera reacted with FaeG1 and FaeG2, respectively, anti-FaeG1 was significantly more prevalent than anti-FaeG2 (*P* < 0.0001). These results were not biased by collection location as sera samples were collected from various regions in Japan (Supporting Tables S1 and S2).

**Table 1.** Prevalence of antibodies against FaeGo, FaeG1 and FaeG2 among porcine and bovine sera

Antigens			Porcine sera (*n* = 200) No. of samples (%)	Bovine sera (*n* = 105) No. of samples (%)	*P* value^a^
FaeG_O_ (K88)	FaeG1 (K88_O169_)	FaeG2 (K88_O169_)			
+	+	+	8 (4.0)	3 (2.9)	*P* = 0.7535
+	+	−	12 (6.0)	0 (0)	*P* = 0.0098
+	−	+	3 (1.5)	1 (1.0)	*P* > 0.9999
−	+	+	3 (1.5)	2 (1.9)	*P* > 0.9999
+	−	−	21 (10.5)	1 (1.0)	*P* = 0.0017
−	+	−	9 (4.5)	8 (7.6)	*P* = 0.2968
−	−	+	1 (0.5)	4 (3.8)	*P* = 0.0495
−	−	−	143 (71.5)	86 (81.9)	*P* = 0.0515

a Fisher's exact test was employed to assess the association between porcine and bovine seroprevalence.

There were no statistical differences in the prevalence of K88_O169_ antibodies reacting to FaeG1 and/or FaeG2 between porcine and bovine sera. Antibodies against FaeG1 were detected in 16.0% (32/200) of pigs and 12.4% (13/105) of cattle and anti-FaeG2 antibodies were found in 7.5% (15/200) of pigs and 9.5% (10/105) of cattle. There were no statistical differences in the prevalence of antibodies to each of FaeG1 and FaeG2 between porcine and bovine sera (FaeG1; *P* = 0.4979, FaeG2; *P* = 0.5199). Anti-FaeG1 single positive sera were higher for cattle (*n* = 8, 7.6%) than pigs (*n* = 9, 4.5%) (*P* = 0.2968) (Table 1). Anti-FaeG2 single positive sera were significantly higher for cattle (*n* = 4, 3.8%; *P* = 0.0495) than pigs (*n* = 1, 0.5%). In contrast, evaluation of the prevalence of antibodies against FaeG of K88ac (hereinafter, FaeG_O_, to reflect its originality), a typical adhesion factor of porcine ETEC, shows that the antibodies to FaeG_O_ were markedly more prevalent among pigs than cattle (Table 1, Fig. 1).

**Fig. 2.**
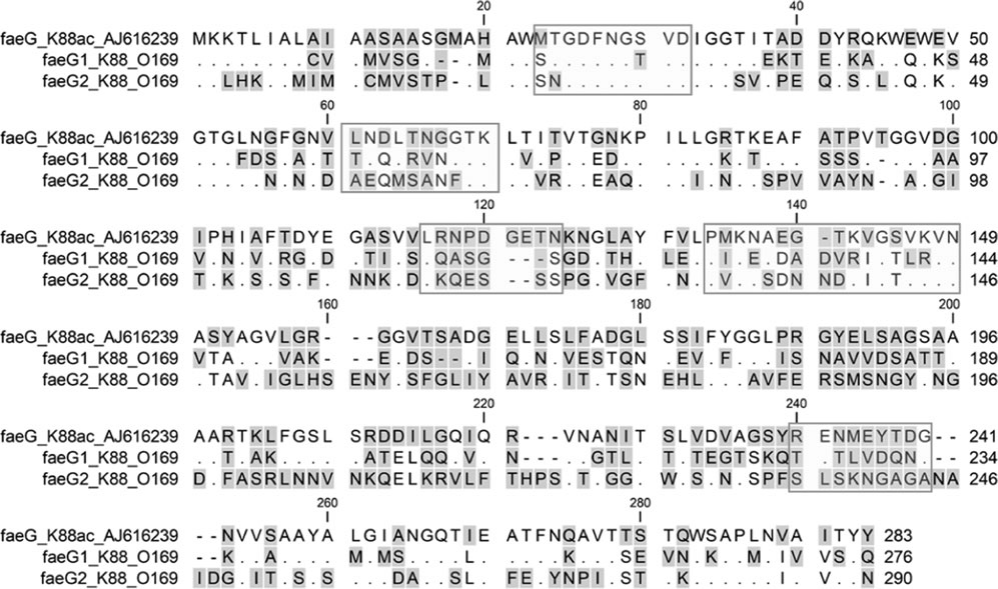
Epitope comparison of FaeG_O_, FaeG1 and FaeG2. The amino acid sequences of FaeG_O_, FaeG1 and FaeG2 were aligned using ClustalW. Residues identical to FaeG_O_ are shown as dots. Hyphens indicate positions where residues are missing. The residue regions enclosed in rectangles are the epitopes referenced in a previous study [15].

**Fig. 3.**
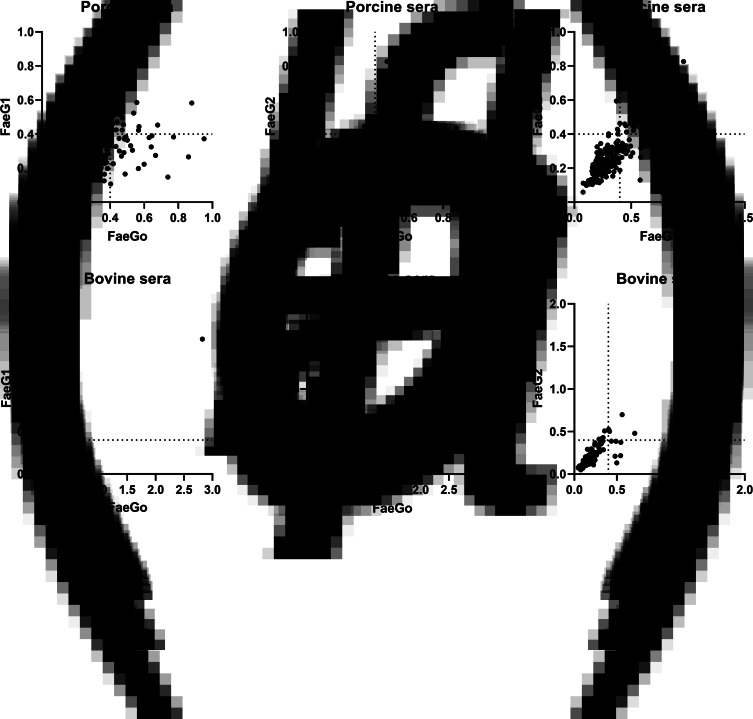
Indirect ELISA plots of FaeG1 or FaeG2 against FaeG_O_. Antigens of FaeG1 or FaeG2 plotted against FaeG_O_ for porcine (a and b) and bovine sera (d and e) show the absence of cross-reaction between each of two antigens. Antibody titres to FaeG1 and FaeG2 showed no relationship to each other in porcine and bovine sera (c and f).

Comparison of epitopes of FaeG_O_, FaeG1 and FaeG2 adhesins shows differences in amino acid sequences (Fig. 2) [15]. Individuals positive for antibodies to FaeG_O_ of K88 did not necessarily cross-react to FaeG1 or FaeG2 of K88_O169_ (Fig. 3). The presence of antibodies to FaeG1 was not associated with the presence of antibodies to FaeG2, and vice versa (Fig. 3). A total of 27 samples were positive for FaeG1 or FaeG2 antigens but not FaeG_O_, and 22 anti-FaeG_O_ positive samples were negative for FaeG1 and FaeG2 antigens (Table 1).

## Discussion

The O169 plasmid pEntYN10 has a total of three CFs (CS6, K88-like and CFA/III-like) [6]. CS6 is recognised as being one of the representative CFs for humans; however, the role of the other two CFs had not yet been elucidated. Since the O169 plasmid can easily be eliminated from bacteria *in vitro* [6], the presence of multiple CFs in the plasmid suggests the possibility that the bacteria can infect various species of hosts consecutively. We used seroepidemiological screening to evaluate whether ETEC possessing K88_O169_ could infect pigs because K88 is a classical CF of porcine ETEC.

Porcine and bovine sera reacted to one or both of FaeG1 and FaeG2 antigens of K88_O169_. These results suggest that K88_O169_-positive *E. coli* might infect pigs and cattle. Although the K88_O169_ operon codes two paralogous major adhesin-like subunits, FaeG1 and FaeG2, FaeG1 seems likely to be the adhesin since anti-FaeG1 was obviously more prevalent than anti-FaeG2. The FaeG_O_ positivity rate was significantly higher among pigs than cattle (*P* < 0.0001), which seroepidemiologically reflects that colonisation of ETEC possessing K88 is more prevalent among pigs. This is consistent with previous reports that the original K88 is a typical adhesion factor of ETEC that causes diarrhoea in piglets [16, 17]. K88ac was used as a positive control antigen, and we consider that these data support the validity of using ELISA in this study.

We considered the possibility that anti-FaeG_O_ cross-reacts with FaeG1 and FaeG2. However, epitopes of FaeG_O_, FaeG1 and FaeG2 adhesins show differences in amino acid sequences. Individuals positive for antibodies to FaeG_O_, FaeG1, or FaeG2 alone were observed in both pigs and cattle. These results suggest that individuals infected with the bacteria that harbour FaeG1, FaeG2, or the homologous CF could be prevalent in these species; positive reactions to these novel antigens are unlikely due to the cross-reaction of antibodies to FaeG_O_.

CF is generally recognised as being the mechanism that determines host specificity. However, our findings showed that anti-K88_O169_ was prevalent among bovine sera and porcine sera, suggesting that K88_O169_ may provide the strain with a mechanism that allows it to infect multiple hosts. The presence of CS8-like CF plasmid, the third and novel one of the O169 strain, may also enhance survival strategies by expanding the host spectrum bacteria in which it is present. Interestingly, virulence plasmid pEntYN10 tended to be quickly eliminated *in vitro* because there are fewer genes associated with plasmid maintenance [6]. In contrast, this plasmid is maintained in ETEC O169 despite this disadvantage; the plasmid likely includes CFs that make it possible for the bacteria to infect multiple hosts, which, in turn, allows the bacterial hosts to retain the plasmid.

Seroepidemiology is an indirect detection method, and the detection of antibodies can not be used to prove that the animal was infected with or that it ever carried the infectious agent being studied. We have not yet isolated these types of bacteria from animals and livestock products. Further, we do not know if the K88_O169_-possessing bacteria are pathogenic or innocuous in animals; specific hosts where the K88_O169_ acts as the CF remain to be identified. Another investigation is ongoing to look for K88_O169_-possessing bacteria in the porcine and bovine faecal matter by PCR using primer sets designed for the genes of the K88_O169_ family.

In conclusion, antibodies to K88_O169_ antigens are prevalent among pigs and cattle. K88_O169_-possessing organisms may expand their niches through a unique and complex repertory of CFs. The present data imply that not only O169 but also other serogroups of *E. coli* possessing K88_O169_ could be transmitted from domestic animals to humans or from humans to domestic animals through the switchover of different adhesins across hosts. We should pay attention to ETEC as a possible pathogen of zoonosis.

## Data Availability

The datasets generated during the current study are available from the corresponding author on reasonable request.

## Supplementary material

For supplementary material accompanying this paper visit http://doi.org/10.1017/S0950268821002697. click here to view supplementary material
